# A STELLA simulation model for *in vitro* dissolution testing of respirable size particles

**DOI:** 10.1038/s41598-019-55164-0

**Published:** 2019-12-06

**Authors:** Basanth Babu Eedara, Ian G. Tucker, Shyamal C. Das

**Affiliations:** School of Pharmacy, University of Otago, 18 Frederick St, Dunedin, 9054 New Zealand

**Keywords:** Respiratory tract diseases, Software

## Abstract

*In vitro* dissolution testing is a useful quality control tool to discriminate the formulations and to approximate the *in vivo* drug release profiles. A dissolution apparatus has been custom-made for dissolution testing of dry powder formulations in a small volume of stationary medium (25 μL spread over 4.91 cm^2^ area i.e. ~50 μm thick). To understand the system and predict the key parameters which influence the dissolution of respirable size particles, a simulation model was constructed using STELLA modeling software. Using this model, the permeation (dissolution followed by diffusion through the membrane) of two anti-tubercular drugs of differing solubilities, moxifloxacin (17.68 ± 0.85 mg mL^−1^) and ethionamide (0.46 ± 0.02 mg mL^−1^), from the respirable size particles and their diffusion from a solution were simulated. The simulated permeation profiles of moxifloxacin from solution and respirable size particles were similar, indicating fast dissolution of the particles. However, the simulated permeation profile of ethionamide from respirable size particles showed slower permeation compared to the solution indicating the slow dissolution of the respirable size particles of ethionamide. The sensitivity analysis suggested that increased mucus volume and membrane thickness decreased the permeation of drug. While this model was useful in predicting and distinguishing the dissolution behaviours of respirable size moxifloxacin and ethionamide, further improvement could be made using appropriate initial parameter values obtained by experiments.

## Introduction

*In vitro* dissolution testing is a standardized quality control tool in all the pharmacopoeias for immediate and controlled release formulations and it is also used to simulate *in vivo* release profiles^1^. However, there is no accepted standardized method to estimate the dissolution profiles of inhaled powder particles; although many dissolution methods (compendial (USP 2) paddle apparatus, flow-through cell apparatus, dialysis bag, Franz diffusion cell, Transwell^®^ and Dissolv*It*^®^ systems) for testing aerosols have been described^2,3^.

Development of an *in vitro* dissolution method which mimics the *in vivo* conditions of the lungs, such as limited volumes of lung fluid (approximately 10–20 mL/100 m^2^)^4^ is challenging. We have custom-made a dissolution apparatus and evaluated the dissolution behaviour of 1–5 µm drug particles in small volumes (25 μL spread over 4.91 cm^2^ area i.e. ~50 μm thick) of stationary simulated mucus fluid^5^. The apparatus is similar to the Dissolv*It*^®^ technology^6^. Development of an *in vitro* dissolution method requires a mechanistic understanding of the system and identification of the key variables controlling the rate of dissolution. In this regard, mathematical models are useful in the scientific understanding of a complex system^7–9^.

Various commercial software packages such as Cloe PK (Cyprotex Ltd., Macclesfield, Cheshire, UK)^10^, GastroPlus (Simulations Plus Inc., California, USA)^11,12^, PulmoSim™ (Pfizer, New York, USA)^13^, MEDICI‐PK (Computing in Technology GmbH, Rastede, Germany)^14^, PKSim/MoBi (Bayer Technology Services, Leverkusen, Germany)^15,16^, SimCyp (Simcyp Ltd., Sheffield, UK)^17^, GI-Sim (AstraZeneca, Cambridge, UK)^18^, MatLab (MathWorks, Inc., Massachusetts, USA)^19^, Berkeley Madonna (University of California, Berkeley, CA, USA)^20^ and STELLA^®^ (Structural Thinking, Experimental learning Laboratory with Animation, isee systems, Lebanon, New Hampshire, USA)^9^ have been used for constructing mathematical models. Among them, PulmoSim™ (developed from SimCyp^®^ by Pfizer) and GastroPlus™ (developed from Simulations Plus) are the commercially available generic tools used to construct physiologically based pharmacokinetic (PBPK) models for inhalable drugs^21^. They have used pharmacokinetic results from laboratory animal (rat) studies together with drug permeability, mucociliary clearance and pulmonary metabolism data to predict the pharmacokinetics of inhaled drugs in humans^22^. However, the complexity of lung physiology and inhaled delivery complicate the development of PBPK models, and it is challenging to include all the processes in a single model.

In this study we aimed to construct a simple STELLA simulation model to predict the dissolution behaviour of respirable size inhaled dry powder particles in a small volume of mucus simulant and diffusion through a membrane in a custom-made dissolution apparatus. STELLA^®^ is user-friendly and icon-based modeling software used to construct a graphical model of a complex system^23^. After construction of a model, the program calculates the values of each variable in the model at each successive time increment using a numerical integration using Euler’s or a Runge-Kutta method^24^. STELLA^®^ has been used effectively to construct a variety of pharmaceutically-related simulation models for: pharmacokinetics of orally administered drugs^25–34^, ocular pharmacokinetics^35^, performance of sustained release dosage forms^36,37^, prodrug performance^38^, and gene expression^39^. The STELLA simulation model described here was used to simulate the permeation (dissolution followed by diffusion through the membrane) of a drug from respirable size particles of two anti-tubercular drugs, moxifloxacin and ethionamide, that have different aqueous solubilities. Further, sensitivity analysis of the model was conducted by varying the volume of the mucus, perfusate flow rate, and membrane thickness to determine their influence on the predicted percentage of drug collected into the collection tubes.

## Methods

### Materials

Materials (suppliers) were as follows: Moxifloxacin (>98% purity) (Leader Biochemical Group Xi’an Leader Biochemical Engineering Co. Ltd., Xi’an, China); ethionamide (≥99.0% purity) (Hangzhou Dayangchem Co., Ltd., Hangzhou, China); polyethylene oxide (Polyox coagulant, molecular weight 5000 kDa, LR grade) (BDH Chemicals Ltd., Poole, England); phosphate buffered saline (Dulbecco A, pH 7.3 ± 0.2; 0.01 M phosphate buffer, 0.003 M potassium chloride and 0.137 M sodium chloride) tablets (Oxoid Ltd., Basingstoke, UK); and dialysis membrane (molecular weight cut off, MWCO, 12,400 Da) (Sigma-Aldrich New Zealand Ltd., Auckland, New Zealand). Freshly collected and filtered (0.45 μm membrane filter) purified water was used.

### *In vitro* dissolution apparatus and method

**Figure 1:**
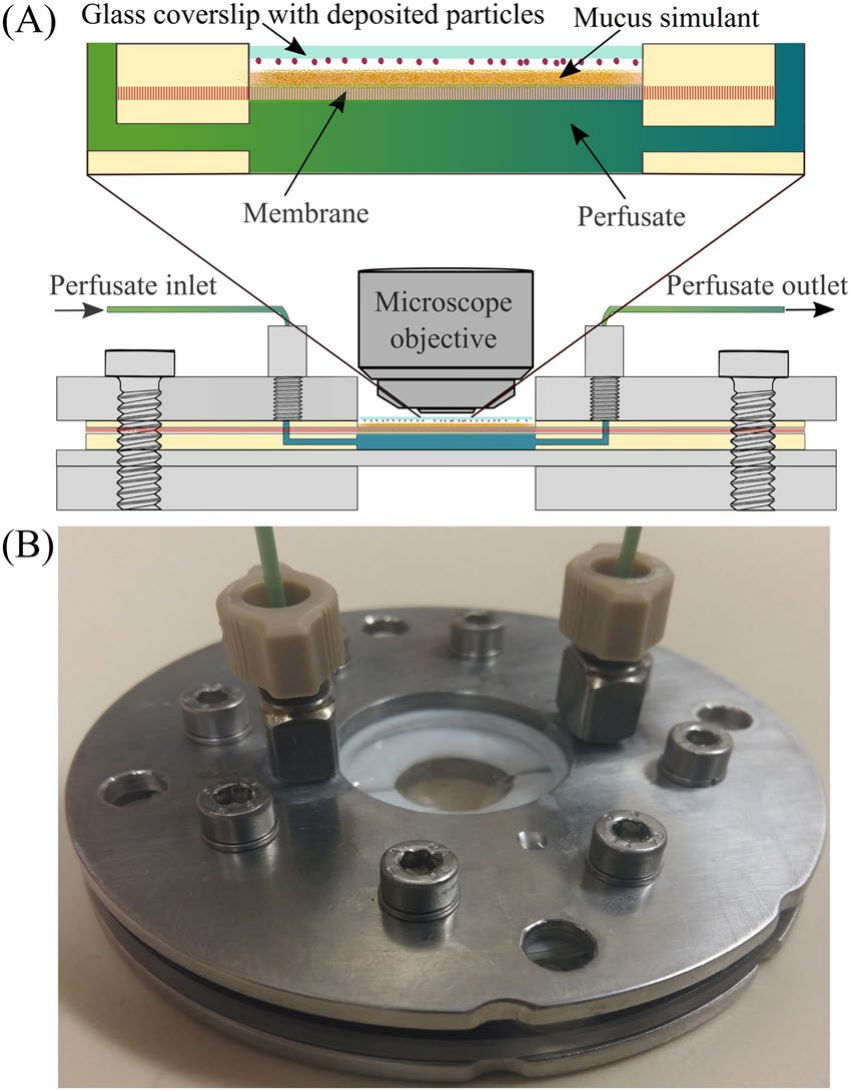
Schematic diagram of the dissolution apparatus (**A**) and custom-made flow perfusion cell (**B**). *Reproduced with permissions from Eedara et al*.^5^.

The custom made dissolution apparatus and dissolution method have been described previously^5,40^. In brief, the apparatus (Fig. 1A; schematic diagram of complete setup) consists of a flow perfusion cell (Fig. 1B) connected to a syringe pump, and an optical microscope equipped with a digital camera.

A membrane is used as a diffusion barrier which allows the transfer of dissolved drug from donor (mucus simulant) to receiver (perfusate) compartment^41^ Ideally, a membrane for *in vitro* dissolution testing of respirable particles should possess the same resistance to drug permeation as the lung epithelium. However, practically, the membrane should not be highly resistive and so that the permeation of dissolved drug becomes the limiting step. It should allow the diffusion of dissolved drug molecules through membrane to evaluate the dissolution rate of respirable size drug particles. In this study, a dialysis membrane (MWCO = 12,400 Da) was selected as this is less resistive and commonly used in drug diffusion studies^42–44^. The thickness of the hydrated dialysis membrane is ∼60 μm which is comparable to the thickness of the ciliated cells in the upper airway region (tracheobronchial region) of the lung^5^.

Phosphate buffered saline (PBS, pH 7.4, 37 °C) was pumped on the blood side of the membrane using the syringe pump. A mucus simulant i.e. polyethylene oxide (PEO) with a MWCO of 5000 kDa was dissolved in PBS (pH 7.4) and used as a dissolution medium as reported by Shah *et al*.^45^. An aliquot of mucus simulant (1.5% w/v polyethylene oxide in PBS, pH 7.4) was uniformly spread over the membrane. The respirable fraction of supplied moxifloxacin and ethionamide particles were collected onto the glass coverslips using a modified Twin Stage Impinger (mTSI; Supplementary Figure S1) and brought into the contact of the mucus simulant applied on the membrane. The particle disappearance was observed using a recording optical microscope. The perfusate samples were collected continuously into the tubes over 120 min, and drug concentrations were quantified by validated HPLC assays as described in our previous publication Eedara *et al*.^5^.

### A STELLA simulation model for *in vitro* dissolution testing of respirable size dry powder particles

**Figure 2:**
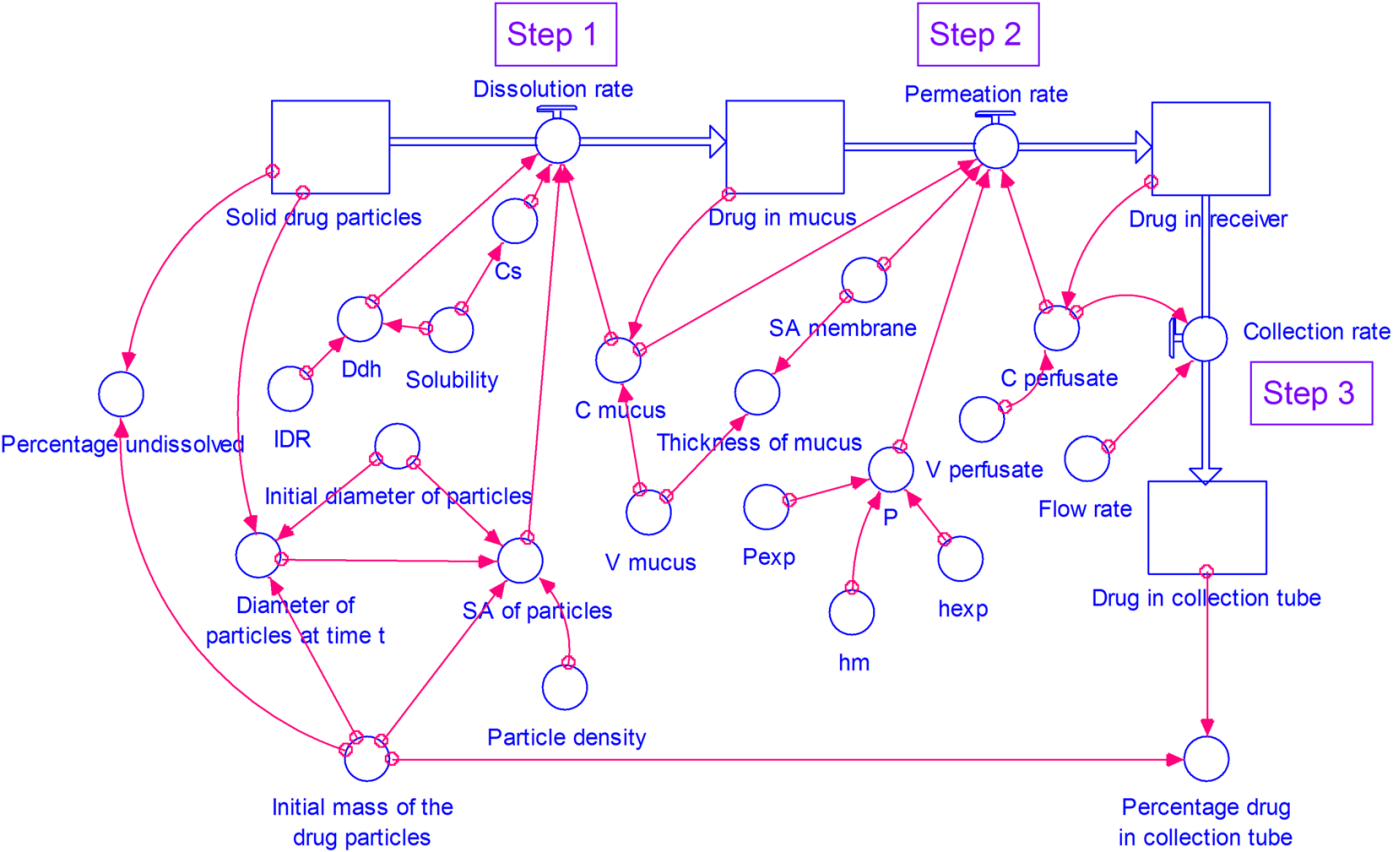
A STELLA model for dissolution of drug from respirable size particles at small volume of mucus simulant under well-stirred conditions. (C- concentration, C_s_- saturation concentration, Ddh- *D/h*, h_exp_- thickness of the membrane used in the experimental study, h_m_- thickness of the membrane, IDR- intrinsic dissolution rate, P- permeability coefficient at time *t*, P_exp_- experimental permeability coefficient, SA- surface area, V- volume).

**Figure 3:**
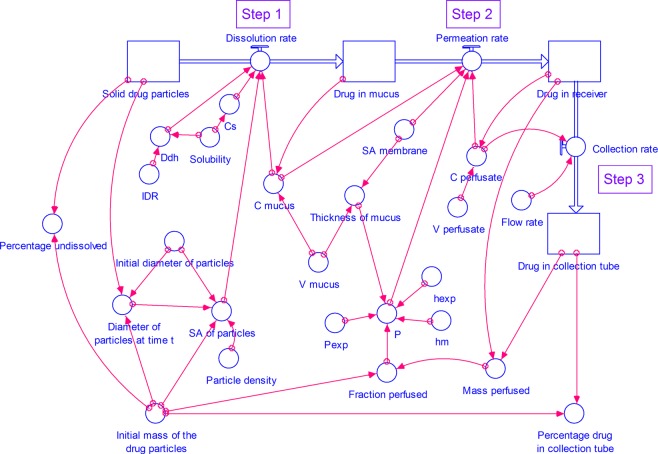
A STELLA model for dissolution of drug from respirable size particles at small volume of mucus simulant under unstirred conditions. (C- concentration, C_s_- saturation concentration, Ddh- D/h, h_exp_- thickness of the membrane used in the experimental study, h_m_- thickness of the membrane, IDR- intrinsic dissolution rate, P- permeability coefficient at time *t*, P_exp_- experimental permeability coefficient, SA- surface area, V- volume).

The model consists of four stocks (compartments) (Figs. 2 and 3) representing drug in solid particulate form, dissolved drug in mucus simulant, permeated drug in receiver and collection tube. All the compartments are connected in a stepwise manner as follows:

- Step 1. Dissolution of respirable size dry powder particles in a small volume of mucus simulant.
- Step 2. Permeation of dissolved drug from mucus simulant through the dialysis membrane representing the lung epithelial membrane into the perfusate in the receiver.
- Step 3. Collection of the perfusate with permeated drug into a collection tube.

These steps are described in detail in the following paragraphs.

***Step 1. Dissolution of respirable size dry powder particles***

In this step (Figs. 2 and 3), the respirable size particles undergo dissolution in the mucus simulant applied on the membrane.

**Figure 4:**
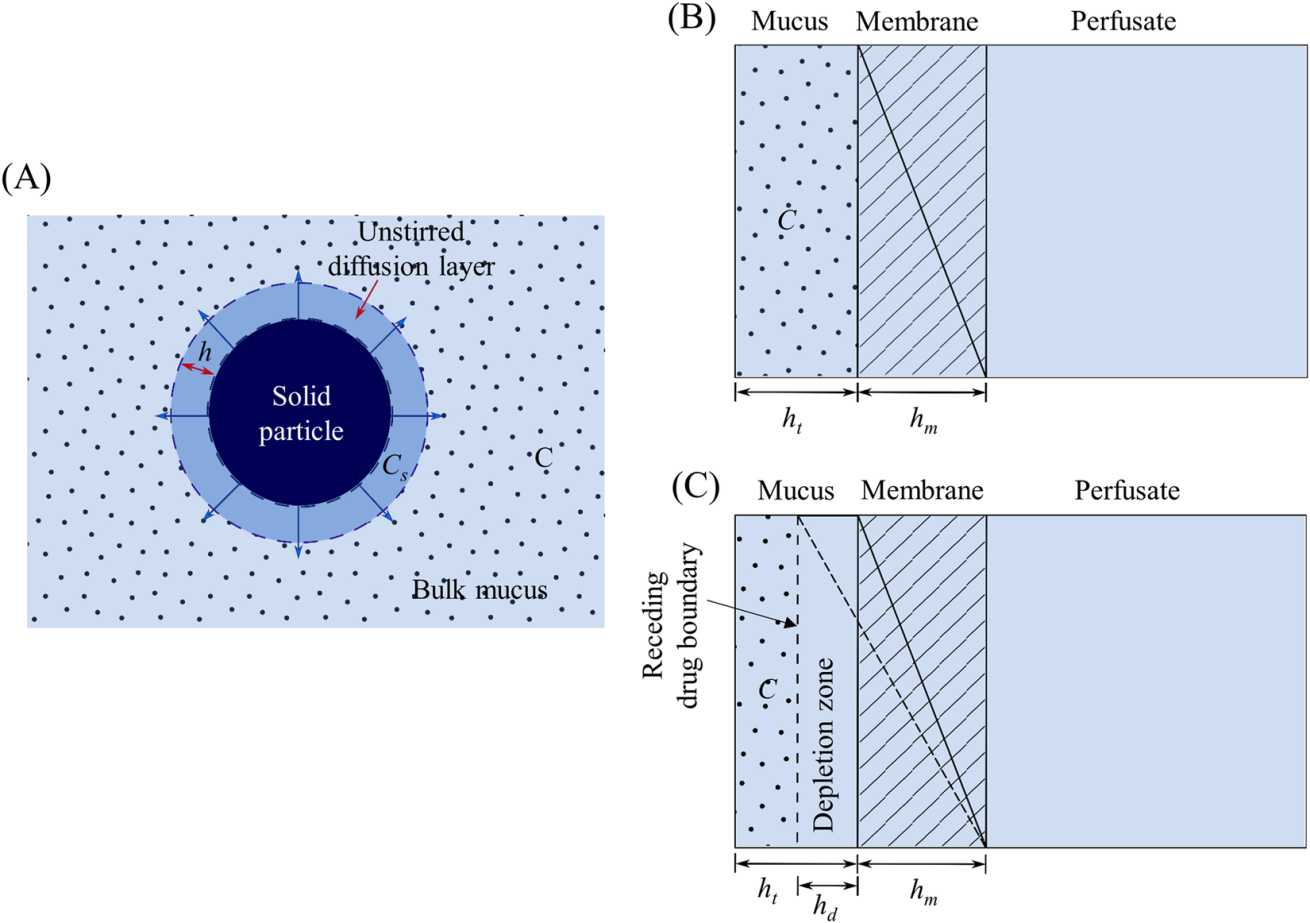
Dissolution of drug particle, showing the unstirred diffusion layer between the particle surface and bulk solution (**A**), and schematic of drug diffusion from mucus through the membrane at well-stirred (**B**) and unstirred condition (**C**) at pseudo steady state. (*C*- concentration of the drug in mucus simulant at time *t*, C_s_- concentration of a saturated solution of the drug, *h*- thickness of the mucus unstirred diffusion layer around each particle, *h*_*d*_- thickness of a depletion zone in the unstirred mucus phase at time *t*, *h*_*m*_- thickness of the membrane, and *h*_*t*_- thickness of mucus).

The dissolution rate of the drug particles in the mucus simulant is calculated using the Noyes-Whitney equation^46^ as shown in Fig. 4A.

1 $$\frac{dM}{dt}=\frac{DS}{h}({C}_{s}-C)$$

In which *M* is the mass of drug dissolved in time *t*, *dM/dt* is the mass rate of dissolution (mass/time), *D* is the diffusion coefficient of the drug in mucus, *S* is the total surface area of the particles at time *t*, *h* is the thickness of the mucus unstirred diffusion layer, *C*_*s*_ is the solubility of the drug i.e. concentration of a saturated solution of the drug at the surface of the solid particle at a given temperature, and *C* is the concentration of the drug in mucus simulant at time *t*.

In Eq. 1, the quantity *D/h* may be referred to as a dissolution rate constant, *k* can be determined from the intrinsic dissolution rate and the solubility of the drug as:

2 $$\frac{D}{h}=k=\frac{Intrinsic\,dissolution\,rate\,(IDR)\,of\,drug}{Solubility\,of\,drug\,({C}_{s})}$$

The concentration of drug in solution in mucus (*C*) is calculated by

3 $$C=\frac{Drug\,in\,solution\,in\,mucus}{Volume\,of\,mucus}$$

The total surface area of the powder at time *t*, *S*_*t(total)*_ is calculated by

4 $$\begin{array}{rcl}Total\,surface\,area\,of\,powder\,at\,time\,t,\,{S}_{t(total)} & = & {S}_{t(particle)}\\  &  & \times \,Number\,of\,particles=\pi {d}_{t}^{2}\\  &  & \times \,\frac{6{M}_{0}}{\pi {d}_{0}^{3}\rho }=\frac{6{M}_{0}{d}_{t}^{2}}{{d}_{0}^{3}\rho }\end{array}$$

5 $$Diameter\,of\,the\,particle\,at\,time\,t,{d}_{t}=\sqrt[3]{\frac{{M}_{t}{d}_{0}^{3}}{{M}_{0}}}$$

In which *M*_0_ and *M*_*t*_ are the masses of the powder at time 0 and *t*, *d*_0_ and *d*_*t*_ are the diameters of a particle at time 0 and *t*, and ρ is the density of the powder. In the model, all particles are assumed to be smooth, spherical and of the same size.

### Step 2. Permeation of dissolved drug through the membrane

In this step, the dissolved drug in the mucus simulant, diffuses through the membrane. We have simulated two models: 1. with a well-stirred mucus phase (See Figs. 2) and 2. an unstirred (See Fig. 3) mucus phase i.e. donor compartment.

Diffusion of drug molecules into the receptor phase is a combination of diffusion through mucus simulant, the membrane, and the unstirred water layers (UWLs, assumed to be of negligible thickness compared to the mucus and membrane) which are present on both sides of the membrane. In a well-stirred system (Fig. 4B), the drug in mucus is homogeneously distributed. Thus, membrane of thickness *h*_*m*_ is the only barrier for diffusion of drug from mucus in a well-stirred system i.e. total diffusion barrier thickness *h*_*T*_ = *h*_*m*_. In an unstirred system (Fig. 4C), as drug passes out of homogeneous mucus, the boundary of drug (dotted line in Fig. 4C) moves towards the left and forms a depletion zone of a thickness *h*_*d*_.

6 $$Thickness\,of\,depletion\,zone\,{h}_{d}=fraction\,of\,drug\,in\,the\,receptor\,({f}_{d})\times thickness\,of\,mucus\,({h}_{t})$$

Thickness of depletion zone increases with increasing drug diffusion. The total diffusion barrier thickness in an unstirred system is the thickness of membrane (*h*_*m*_) and depletion zone (*h*_*d*_) i.e. *h*_*T*_ = *h*_*m*_ + *h*_*d*_ = *h*_*m*_ + *f*_*d*_ × *h*_*t*_.

The permeation rate of the drug through a well-stirred or unstirred system was calculated as follows

7 $$Permeation\,rate=PS({C}_{mucus}-{C}_{perfusate})$$

Where permeability coefficient *P* (cm s^−1^) is defined as the diffusion coefficient *D* multiplied by the partition coefficient *K*, and divided by the diffusional thickness, *h* and S is the surface area of the membrane. There is no partitioning of drug involved in the case of drug diffusion through the mucus and membrane with aqueous pathways. Thus, P can be expressed as

8 $$P=\frac{D}{h}$$

In general, it is difficult to determine the *D* and *h* independently in order to calculate *P*. However, permeability coefficient can be measured experimentally (*P*_*exp*_) by measuring the rate of permeation and knowing *S*, concentration of drug in the donor phase (mucus) *C*_*d*_ and volume of donor phase (mucus) *V*_*d*_. It can be obtained from the slope of ln C_d_ versus time *t*:

9 $$\mathrm{ln}\,{C}_{d}=\,\mathrm{ln}\,{C}_{d0}-\frac{PSt}{{V}_{d}}$$

10 $${P}_{exp}=\frac{D}{{h}_{exp}}$$

Where *h*_*exp*_ is the thickness of the membrane used in the experimental study.

In a well-stirred system, the thickness of the diffusion barrier remains constant throughout the diffusion process. Thus, the permeability coefficient also remains constant (*P*_*exp*_).

In a well-stirred system, at all times, *h*_*T*_ = *h*_*m*_

11 $$P=\frac{D}{{h}_{T}}=\frac{D}{{h}_{m}}$$

By substituting the experimental permeability coefficient (i.e. Eq. 10) in Eq. 11

12 $$P=\frac{{P}_{exp}{h}_{exp}}{{h}_{m}}$$

In an unstirred system the thickness of the diffusion barrier increases with increasing diffusion due to formation of the depletion zone; hence the permeability coefficient varies with time.

In an unstirred system, at time 0, *h*_*T*_ = *h*_*m*_

13 $${P}_{0}=\frac{D}{{h}_{T}}=\frac{D}{{h}_{m}}$$

At time *t*, *h*_*T*_ = *hm* + *f*_*d*_ × *h*_*t*_

14 $${P}_{t}=\frac{D}{{h}_{T}}=\frac{D}{{h}_{m}+{f}_{d}{h}_{t}}$$

By substituting experimental permeability coefficient (i.e. Eq. 10) in Eq. 14 the permeability coefficient at time *t* is:

15 $${P}_{t}=\frac{{P}_{exp}{h}_{exp}}{{h}_{m}+{f}_{d}{h}_{t}}$$

### Step 3. Collection of the perfusate containing permeated drug into a collection tube

In this step (See Figs. 2 and 3), perfusate is continuously removed from the receiver into the collection tubes. Thus, the drug permeated into the perfusate in the receiver compartment is transferred into the collection tubes at a rate calculated using Eq. 16.

16 $$Collection\,rate=Concentration\,of\,drug\,in\,perfusate\times Perfusate\,flow\,rate$$

#### The components of the model

Step 1 - dissolution of respirable size dry powder particles in small volume of mucus simulant, Step 2 - permeation of dissolved drug from mucus through the dialysis membrane, and Step 3 - collection of the perfusate with permeated drug into a collection tube are linked to construct a model for dissolution of drug from respirable size particles at small volume of mucus simulant under well-stirred (Fig. 2) or unstirred (Fig. 3) conditions. Various equations and assumptions that were made in the construction of the simulation models are described in the Supplementary information (Sections S1 and S2).

### Simulation of the percentage cumulative drug permeated versus time profiles using STELLA simulation model

The simulations for the permeation (dissolution followed by diffusion of drug) of anti-tubercular drugs, moxifloxacin and ethionamide from respirable size particles were conducted using the initial parameter values shown in Table 1. The parameter values used were obtained from the experimental results shown in Eedara *et al*.^5^. The moxifloxacin and ethionamide respirable size particles were assumed to possess a particle density of 1.0 g cm^−3^ ^47^.

**Table 1:** Initial parameter values used for simulation.

Parameters	Moxifloxacin	Ethionamide
Drug solubility (at donor medium pH)^*^	17.70 × 10^−3^ g cm^−3^	0.46 × 10^−3^ g cm^−3^
Intrinsic dissolution rate (at donor medium pH)	0.50 × 10^−3^ g cm^−2^ min^−1^	0.06 × 10^−3^ g cm^−2^ min^−1^
Diameter of particles	2.9 × 10^−4^ cm	3.6 × 10^−4^ cm
Particle density (assumed)	1.0 g cm^−3^	1.0 g cm^−3^
Experimental permeability coefficient (P_exp_)	1.8 × 10^−4^ cm min^−1^	5.3 × 10^−4^ cm min^−1^
Mucus simulant	1.5% w/v PEO in PBS, pH 7.4	
Mucus simulant volume	25 × 10^−3^ cm^3^	
Perfusate, its pH and flow rate	PBS, pH 7.4, 0.4 cm^3^ min^−1^	
Perfusate volume in the receptor	500 × 10^−3^ cm^3^	
Area of the membrane	4.91 cm^2^	
*h*_*exp*_^†^	62.5 × 10^−4^ cm (hydrated membrane)	
*h*_*m*_ (assumed)^†^	62.5 × 10^−4^ cm	

*Drug solubility was measured in PBS, pH 7.4 and we assumed the same solubility in mucus simulant (1.5% w/v polyethylene oxide (PEO) in PBS, pH 7.4) used in the experimental study.

^†^*h*_*exp*_- thickness of the membrane used in the experimental study, *h*_*m-*_ thickness of the membrane used in the STELLA simulations.

### Sensitivity analysis of the model

Sensitivity analyses of the models were conducted by varying the volume of the mucus (25, 50, and 100 μL), perfusate flow rate (0.2, 0.4, and 0.8 mL min^−1^) and membrane thickness (experimental membrane thickness (*h*_*exp*_) 60, 80, 100 μm, and membrane thickness used in the simulation (*h*_*m*_) 30, 60 and 90 μm). The parameters were varied one at a time while keeping others fixed to determine the influence of the parameters on the predicted percentage drug collected into the collection tubes.

## Results and Discussion

### Permeation of drug from solution through the membrane

STELLA models were constructed for permeation of the drug from a solution (only diffusion of drug) through the membrane under well-stirred (Supplementary Figure S2) or unstirred conditions (Supplementary Figure S3). Various equations and assumptions that were made in the construction of the simulation models (Supplementary Figures S2 and 3) are described in the Supplementary information (Sections S3 and S4). The simulations for the permeation of moxifloxacin and ethionamide from solutions were conducted using the initial parameter values shown in Supplementary Table S1.

**Figure 5:**
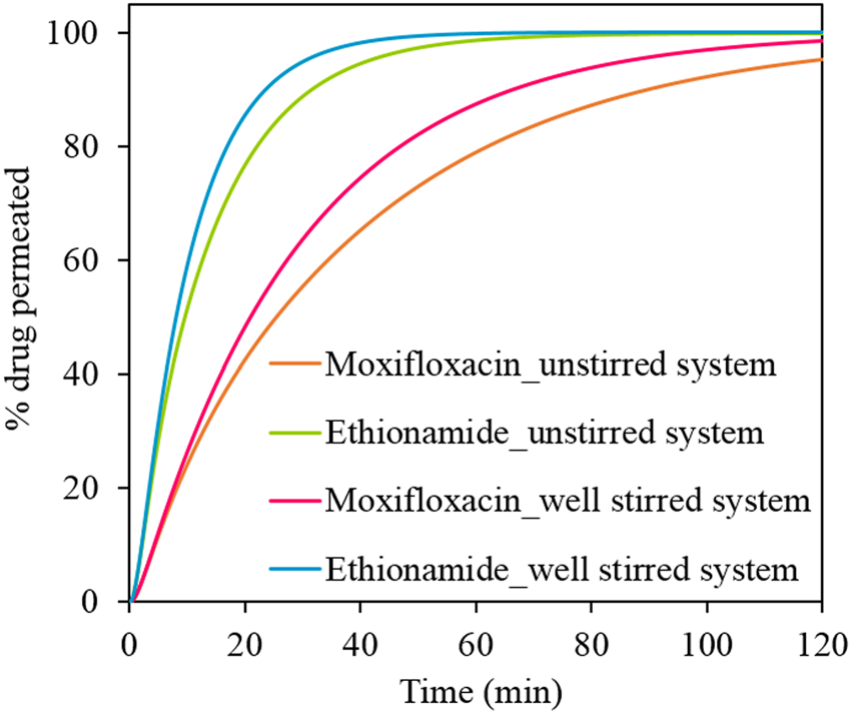
STELLA simulated permeation profiles of moxifloxacin and ethionamide from the solutions (only diffusion of drug) at 25 μL of mucus assuming a homogenous (well-stirred) donor and an unstirred donor with a developing depletion zone.

The experimental permeability coefficient calculated from data covering over 80% permeation for ethionamide (5.3 × 10^−4^ cm min^−1^) is three times higher than that of higher molecular weight moxifloxacin (1.8 × 10^−4^ cm min^−1^)^5^. The simulation profiles (Fig. 5) also show the faster permeation of ethionamide compared to moxifloxacin at both well-stirred and unstirred conditions. Ethionamide showed complete permeation by 60 min at both well-stirred and unstirred conditions. However, moxifloxacin showed slower permeation taking more than 120 min to completely permeate the drug through the membrane. The slower permeation of both moxifloxacin and ethionamide at unstirred condition was due to an increased diffusion barrier thickness with a depletion zone (Fig. 4C).

### Dissolution of drug from the respirable size particles

**Figure 6:**
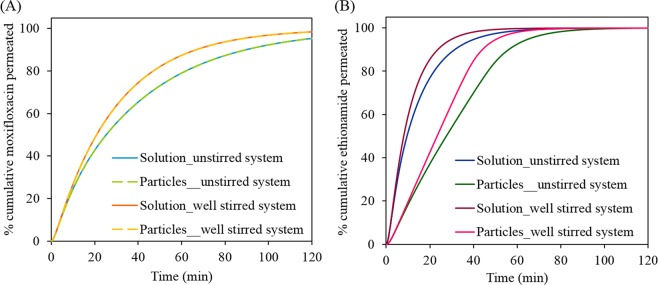
STELLA simulated permeation profiles of moxifloxacin (**A**) and ethionamide (**B**) from the solutions (50 µg in 25 µL of mucus simulant) and respirable size particles (50 µg) under well-stirred and unstirred conditions. The mucus simulant volume and perfusate flow rate were 25 µL and 0.4 mL min^−1^, respectively. In figure (**A**), the moxifloxacin permeation profiles from solution and respirable size particles were superimposed with each other at both well-stirred and unstirred conditions.

Figure 6 shows the simulated permeation profiles of moxifloxacin and ethionamide from solutions of the drugs and from suspensions of respirable size particles using 25 μL of mucus simulant and 0.4 mL min^−1^ perfusate flow rate. The permeation profiles of moxifloxacin from the suspension (dissolution followed by diffusion) and a solution (only diffusion) were superimposable at both well-stirred and unstirred conditions indicating rapid dissolution of the moxifloxacin particles with permeation rate being controlled by the diffusion through the membrane. Overall, ethionamide permeated faster than moxifloxacin; however, the ethionamide respirable particles showed a significant decrease in the permeation of drug compared to the solution at well-stirred and unstirred conditions due to the slow dissolution of poorly water-soluble (0.46 mg mL^−1^)^5^ ethionamide particles in the small volume (25 μL) of mucus simulant. Both moxifloxacin and ethionamide respirable particles showed a faster permeation of drug under the well-stirred condition compared to the unstirred condition as expected.

### Comparison of the experimental permeation profiles with STELLA simulated permeation profiles

**Figure 7:**
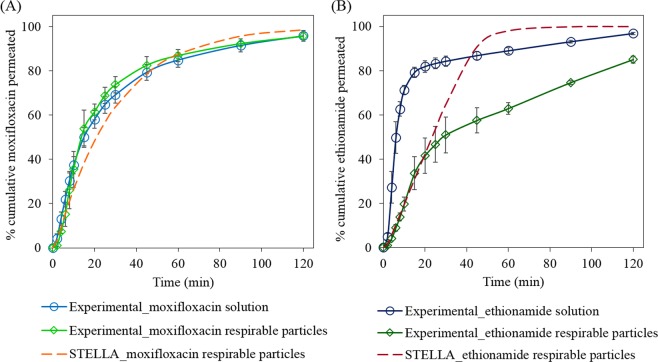
Experimental permeation profiles (solid lines with markers and error bars) of moxifloxacin (**A**) and ethionamide (**B**) from the solutions and respirable size particles (~50 µg) in comparison to the simulation permeation profiles (dashed lines) from respirable size particles under well-stirred condition. The mucus simulant (1.5% w/v PEO in PBS) volume and perfusate (PBS, pH 7.4) flow rate were 25 µL and 0.4 mL min^−1^, respectively. Data are means ± SD (n = 3).

The experimental permeation profiles of moxifloxacin (Fig. 7A) from its solution and respirable particles were not significantly (p > 0.05) different which indicates rapid dissolution of moxifloxacin, and its permeation was limited by diffusion through the membrane. The experimental permeation profile (solid lines with markers and error bars in Fig. 7A) of moxifloxacin from respirable size particles approximately fits with the simulated permeation profiles at well-stirred condition (dashed lines in Fig. 7A). In the case of ethionamide (Fig. 7B), the experimental permeation profile from respirable size particles only fits with the well-stirred STELLA simulated permeation profile over initial 30 min, followed by a slower permeation of the drug.

The differences between the STELLA simulations and the experimental data exemplify the value of simulations, since they indicate that the mechanism incorporated into the existing STELLA model are insufficient to describe the dissolution. Thus, the differences stimulate creative thinking about other factors that may influence drug delivery rate from respirable drug particles. Some possible reasons for the differences in the simulated and experimental permeation profiles of ethionamide are:

1. In the STELLA simulation, the particles are assumed to be smooth, spherical and of uniform size, but the experimental ethionamide powder particles were broken, non-spherical and with a size distribution of 1.5 to 8 µm.
2. The intrinsic dissolution rate (IDR) of the drug used in STELLA simulation was determined in PBS, pH 7.4 as dissolution medium using a compressed disc (1.3 cm^2^) of powder by the rotating disc method. However, during the experimental dissolution study for the respirable size particles, the particles collected using the mTSI are well-separated and smaller in size compared to the compressed powder disc used in IDR study and might possess the unstirred diffusion layer of varying thickness compared to the compressed disc used in the IDR study. Further during the experimental dissolution study, a viscous mucus simulant composed of PEO and PBS, pH 7.4 was used as a dissolution medium in which the diffusion of the dissolved drug might be slower compared to the PBS, pH 7.4 alone used in the IDR study.

These possible reasons provide the basis for other experiments as well as modifications to the STELLA model to achieve a closer agreement between the simulation profiles and the experimental results.

### Sensitivity analysis

Sensitivity analyses of the simulations to the changes in the mucus simulant volume, perfusate flow rate and membrane thickness are shown in following sections.

### Sensitivity to the change in mucus simulant volume

**Figure 8:**
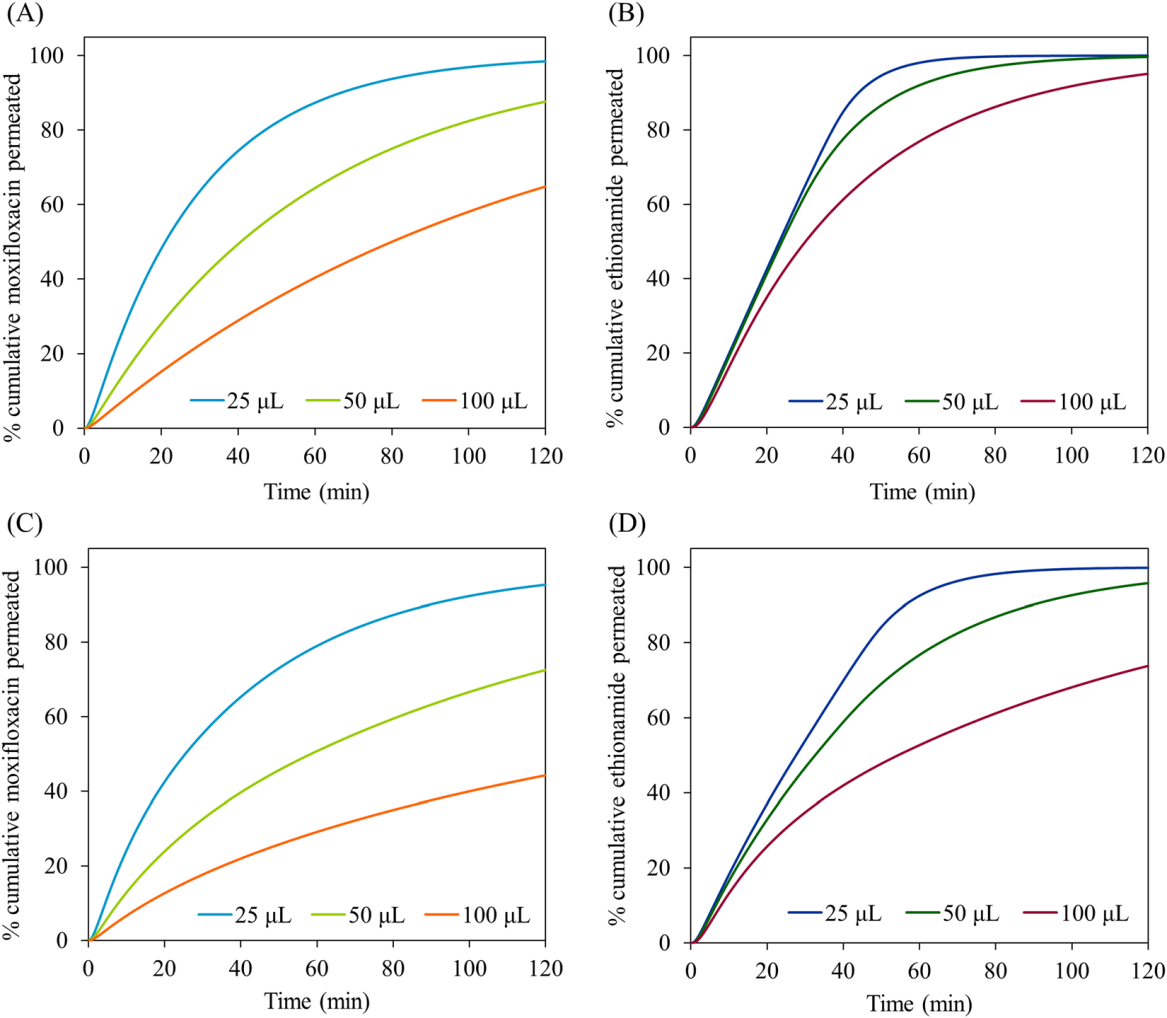
Influence of mucus simulant volume (25, 50 and 100 µL) on the simulated permeation (dissolution followed by diffusion) of moxifloxacin (A- well-stirred condition; C- unstirred condition) and ethionamide (B- well-stirred condition; D- unstirred condition) from the respirable size particle suspensions at 0.4 mL min^−1^ perfusate flow rate under well-stirred condition and unstirred conditions.

Figure 8 shows the simulated permeation profiles of moxifloxacin and ethionamide with increasing mucus simulant volumes (25, 50 and 100 μL) at 0.4 mL min^−1^ perfusate flow rate under well-stirred and unstirred conditions. The permeation of both the drugs decreased with increasing mucus simulant volume at well-stirred and unstirred conditions.

For moxifloxacin all three volumes (25, 50 and 100 μL) of the mucus simulant can solubilize the mass of the moxifloxacin (50 μg) completely, so the concentration of the moxifloxacin in mucus simulant decreases with increasing volumes (25 μL- 2.0 mg mL^−1^ >50 μL- 1.0 mg mL^−1^ > 100 μL- 0.5 mg mL^−1^). Thus the driving concentration in the donor decreases so the permeation rate reduces. For ethionamide, the situation is more complex. The solubility of ethionamide in PBS (0.46 ± 0.02 mg mL^−1^) is much lower that that of moxifloxacin and there is excess undissolved ethionamide in all three volumes of mucus simulant tested (25 μL- 11.5 μg, 50 μL-23.0 μg, 100 μL- 46 μg) at earlier times. Eventually, sufficient ethionamide has permeated such that there is no excess undissolved drug in the donor. This occurs at an earlier time when the donor volume is large. Hence the driving concentration decreases leading to a fall in the permeation rate at earlier times as the donor volume is increased. This result has implications for understanding the absorption rate of drug from the lungs. As for the biopharmaceutical classification system for orally administered drugs, a similar system can be envisaged for the pulmonary route in which drug dose, drug solubility and volume of lung fluid will influence absorption rate.

Under unstirred condition, not only do the above factors operate, but in addition there is an increase in the diffusion (depletion layer) thickness that further slows the permeation of both moxifloxacin and ethionamide with increasing mucus simulant volumes.

### Influence of perfusate flow rate

**Figure 9:**
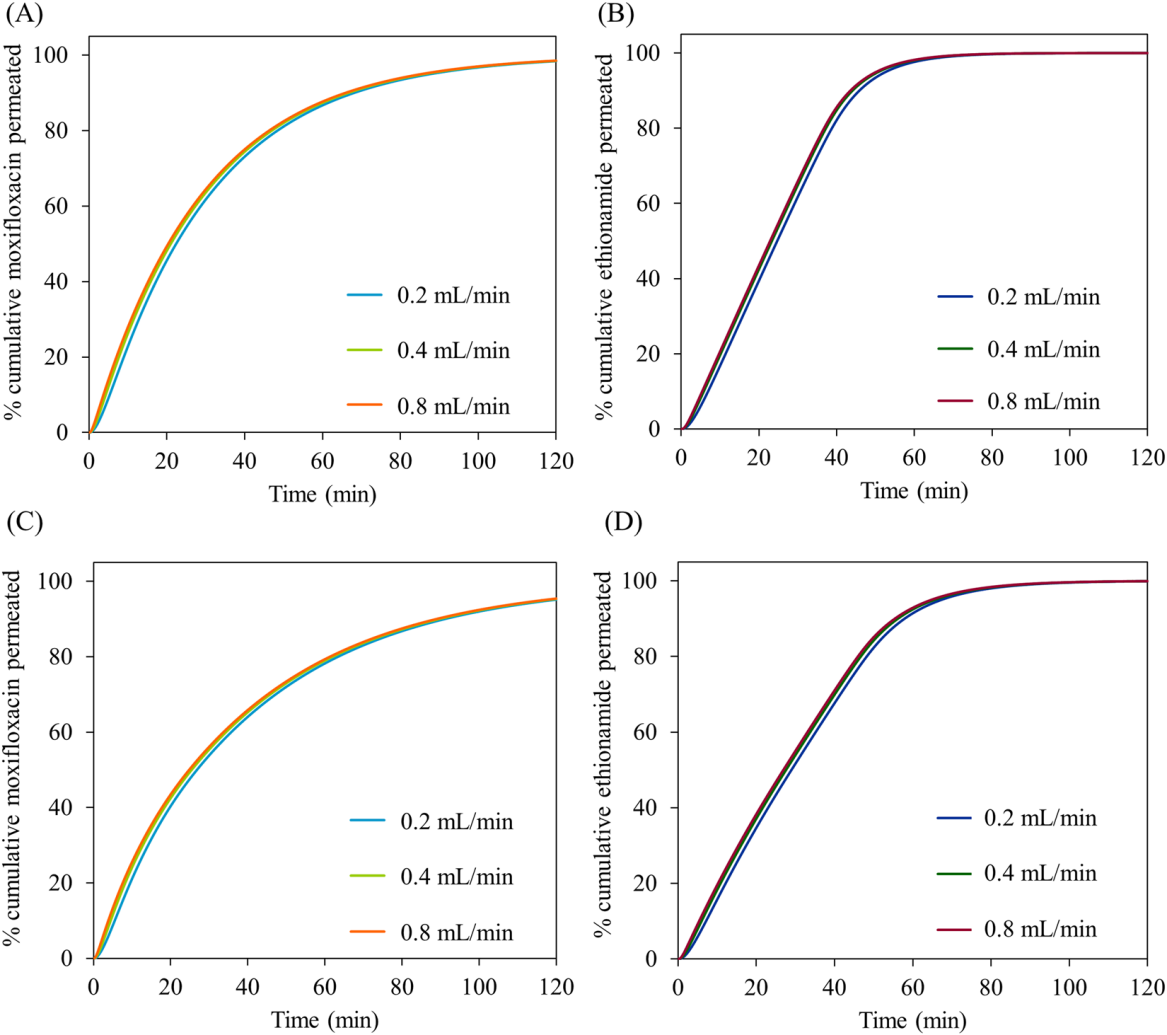
Influence of perfusate flow rate (0.2, 0.4 and 0.8 mL min^−1^) on the simulated permeation (dissolution followed by diffusion) of moxifloxacin (A- well-stirred condition; C- unstirred condition) and ethionamide (B- well-stirred condition; D- unstirred condition) from the respirable particles using 25 µL of mucus.

Figure 9 shows that there was no significant change in the simulated permeation profiles of moxifloxacin and ethionamide from respirable size particles in 25 μL of mucus simulant at increasing perfusate flow rates (0.2, 0.4 and 0.8 mL min^−1^) under well-stirred and unstirred conditions.

The perfusion flow rate could potentially affect permeation rate for two reasons: (i) Its effect on the concentration in the receptor phase and (ii) Its effect on the thickness of the static diffusion layer on the receptor side of the membrane.

Passive diffusion of drug molecules from a solution through the membrane occurs from the region of higher to the lower concentration and is directly proportional to the concentration difference (*C*_*mucus*_* − C*_*perfusate*_) between the donor (mucus) and receptor (perfusate). The calculated concentrations of moxifloxacin and ethionamide in mucus and perfusate at various perfusate flow rates (0.2, 0.4, and 0.8 mL min^−1^) in the STELLA simulation under well-stirred and unstirred condition are presented in Supplementary information (moxifloxacin: at well-stirred condition- Supplementary Table S2, at unstirred condition- Supplementary Table S3, ethionamide: at well-stirred condition- Supplementary Table S4, at unstirred condition- Supplementary Table S5). The perfusate concentrations of both the drugs at all the perfusate flow rates under well-stirred and unstirred conditions were negligible compared with the mucus concentration (*C*_*perfusate*_ ≪ *C*_*mucus*_ *i.e*. (*C*_*mucus*_ *−* *C*_*perfusate*_) ≈ *C*_*mucus*_). Thus, the diffusion rate is proportional to the concentration of the drug in the mucus, *C*_*mucus*_ and thus the perfusate flow rate has no significant effect on the permeation rates of moxifloxacin and ethionamide.

The diffusion barrier consists of mucus simulant, the membrane and the unstirred water layers which are present on both sides of the membrane^48^. The thickness of the unstirred water layers is unknown and might be small compared to the mucus simulant and membrane thickness. Thus, we assumed the thicknesses of mucus simulant and hydrated membrane as the diffusion barrier thickness in the STELLA simulation model. However, with increasing perfusate flow rate, the thickness of the unstirred water layer present beneath the membrane towards the receptor compartment might decrease leading to faster permeation of drug^49^ but this was not built into the model.

### Influence of the membrane thickness

**Figure 10:**
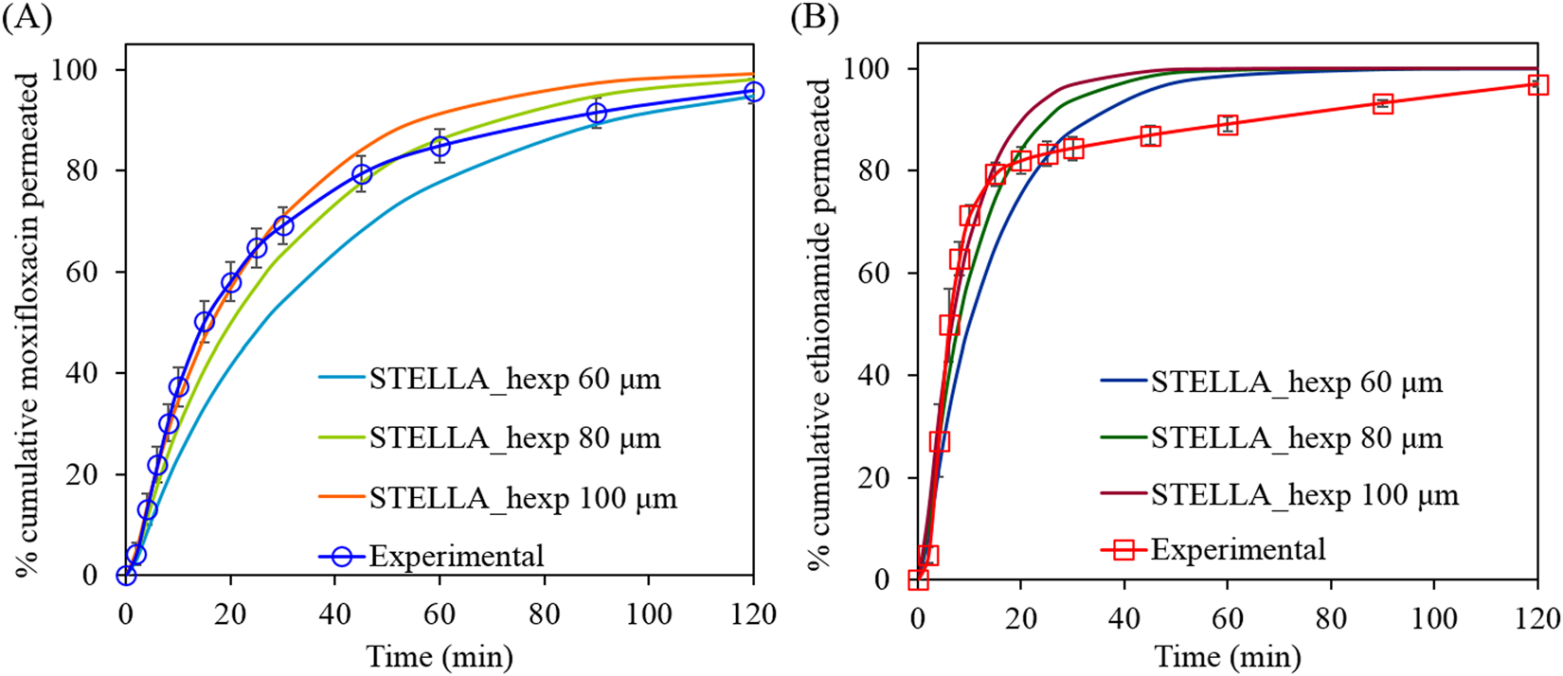
Comparison of the simulated permeation profiles of moxifloxacin (**A**) and ethionamide (**B**) from the solutions (50 µg of moxifloxacin or 10 μg of ethionamide in 25 µL of mucus simulant, 0.4 mL min^−1^ perfusate flow rate) at an experimental membrane thickness (*h*_*exp*_) of 60, 80, and 100 μm under unstirred conditions with the experimental permeation profiles.

The thickness (*h*_*exp*_) of the hydrated membrane used in the experimental drug permeation study was measured using a micrometer. However, the permeation barrier consists of the hydrated membrane plus unstirred water layers on each side of the membrane so the exact thickness of the diffusion barrier is unknown. Thus, we have simulated the permeation profiles (Fig. 10) of moxifloxacin and ethionamide from the solutions (50 µg of the moxifloxacin or 10 μg of ethionamide in 25 µL of mucus simulant, 0.4 mL min^−1^ perfusate flow rate) for a range of experimental membrane thicknesses (*h*_*exp*_ = 60, 80 and 100 μm), and compared the results with the experimental permeation profile to estimate the thickness of the unstirred water layer thickness. As expected, increasing *h*_*exp*_ increase the permeation rate (see Eq. 15) and the simulation profile at *h*_*exp*_ = 100 μm for both moxifloxacin and ethionamide closely matched the experimental permeation profile over 80% of drug permeation. This suggests that the thickness of the unstirred water layer in the receptor was approximately 40 μm.

The simulated and experimental permeation profiles (Fig. 10) of moxifloxacin and ethionamide show an initial near linear steady state permeation followed by a falling permeation rate over 80% drug release due to the decreasing concentration of drug in the donor compartment. However, the experimental permeation profiles of ethionamide show significantly slower permeation after 80% drug release compared to the simulated permeation profiles. The possible reason for the slower permeation of ethionamide might be due to the slower dissolution of larger particles (size distribution of 1.5 to 8 µm) of ethionamide during experimental study.

**Figure 11:**
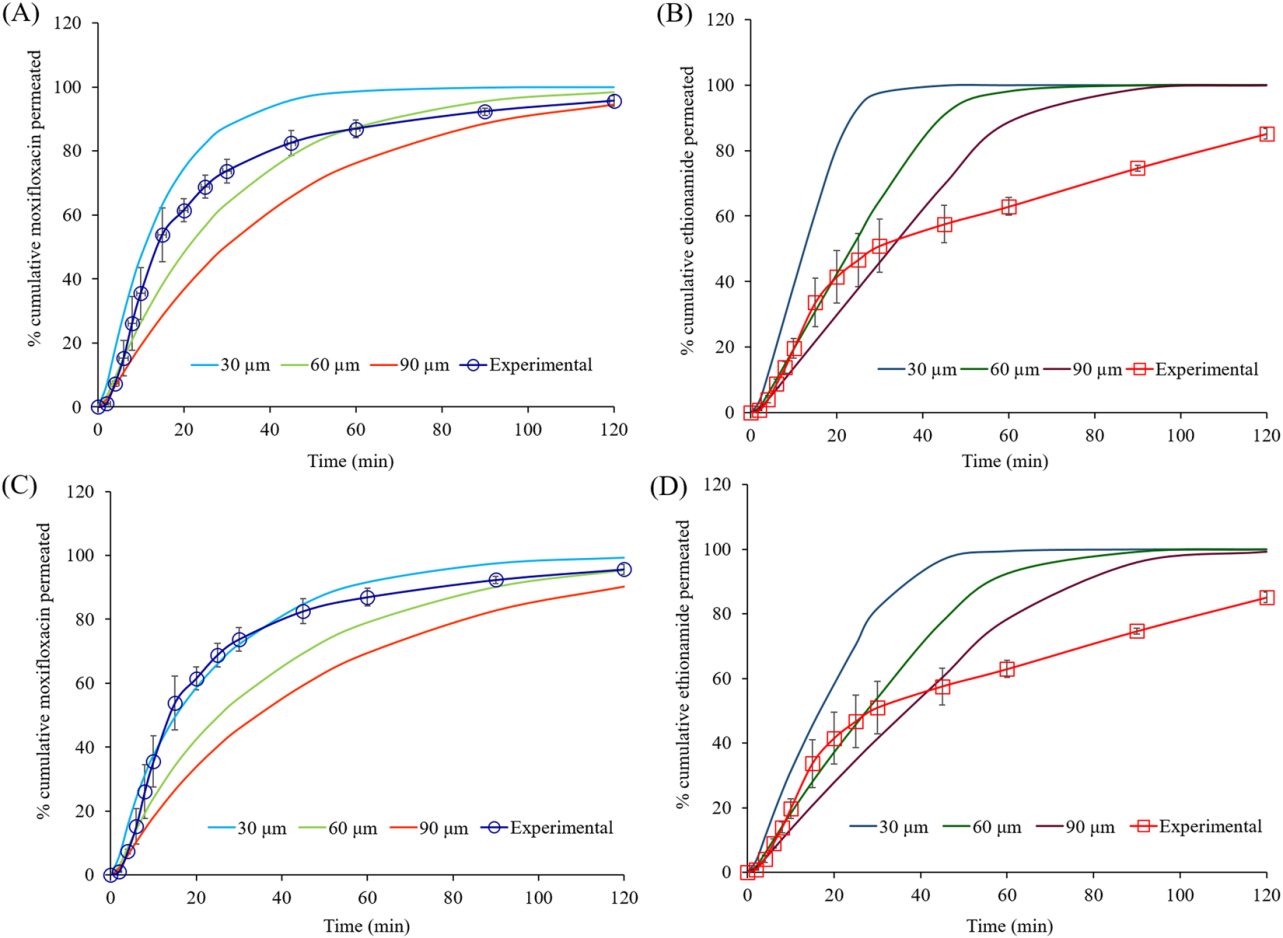
Influence of membrane thickness (30, 60, and 90 μm) on the simulated permeation (dissolution followed by diffusion) of moxifloxacin (A- well-stirred condition; C- unstirred condition) and ethionamide (B- well-stirred condition; D- unstirred condition) from the respirable particles using 25 µL of mucus under well-stirred and unstirred conditions. Experimental permeation profiles (solid lines with markers and error bars) of moxifloxacin and ethionamide from the respirable size particles (~50 µg) using 25 µL of mucus at 0.4 mL min^−1^ perfusate flow rate.

Figure 11 shows the simulated permeation profiles of moxifloxacin and ethionamide from respirable size particles in 25 μL of mucus simulant at increasing membrane thickness (*h*_*m*_) (30, 60, and 90 μm) and 0.4 mL min^−1^ perfusate flow rates under well-stirred and unstirred conditions. With increasing membrane thickness, the diffusional length for the solute molecules increases and leads to a slower permeation. Accordingly, the permeation of both the drugs decreased with increasing membrane thickness (*h*_*m*_) at well-stirred and unstirred conditions. However, the simulated permeation profile of ethionamide was not a close fit to the experimental permeation profile.

## Conclusions

A STELLA simulation model was constructed for *in vitro* dissolution testing of respirable size particles in a custom-made dissolution apparatus. Both the simulations and the experimental data stimulate thinking on operative mechanisms involved in drug delivery via the lungs. The simulated permeation profile of ethionamide from a solution (only membrane diffusion) showed faster permeation compared to moxifloxacin under well-stirred and unstirred conditions. However, the permeation of both the drugs was slower in the model which assumed unstirred conditions as the diffusion layer thickness increases by the formation of a depletion zone under unstirred conditions. The simulated permeation profiles of moxifloxacin from solution (only diffusion) and respirable size particles (dissolution followed by the diffusion through the membrane) were similar, indicating fast dissolution of the particles. However, the simulated permeation profile of the lower soluble ethionamide showed slower permeation compared to the solution indicating that dissolution rate delayed permeation. Increasing the volume of the mucus simulant reduced the permeation rate of moxifloxacin to a greater extent that ethionamide. This suggests the need for a biopharmaceutical classification system for drugs delivered via the lungs and better understanding of lung fluid volumes. Membrane thickness decreased the permeation of drug. This model will be useful to inform understanding of the dissolution behaviour of drug from respirable size particles before proceeding to experimental studies in the custom-made dissolution apparatus.

## Supplementary information

Supplementary information
