# Supplementary material for: A STELLA simulation model for in vitro dissolution testing of respirable size particles
Source: Sci Rep. 2019 Dec 6;9:18522. doi: 10.1038/s41598-019-55164-0 (PMC6898627; doi:10.1038/s41598-019-55164-0)
Supplement: Supplementary file 1 — Supplementary information [file 41598_2019_55164_MOESM1_ESM.docx]

**A STELLA simulation model for *in vitro* dissolution testing of respirable size particles**

**Basanth Babu Eedara, Ian G. Tucker^*^, Shyamal C. Das^*^**

School of Pharmacy, University of Otago, 18 Frederick St, Dunedin 9054, New Zealand.

**^*^Corresponding Authors**

Professor Ian G. Tucker

School of Pharmacy, University of Otago, Adams Building,

18 Frederick Street, P.O. Box 56, Dunedin 9054, New Zealand.

Tel.: +64 3 479 7296; Fax: +64 3 479 7034.

E-mail address: [ian.tucker@otago.ac.nz](mailto:ian.tucker@otago.ac.nz)

Dr Shyamal C. Das

School of Pharmacy, University of Otago, Adams Building,

18 Frederick Street, P.O. Box 56, Dunedin 9054, New Zealand.

Tel.: +64 3 479 4262; Fax: +64 3 479 7034.

E-mail address: [Shyamal.das@otago.ac.nz](mailto:Shyamal.das@otago.ac.nz)

**
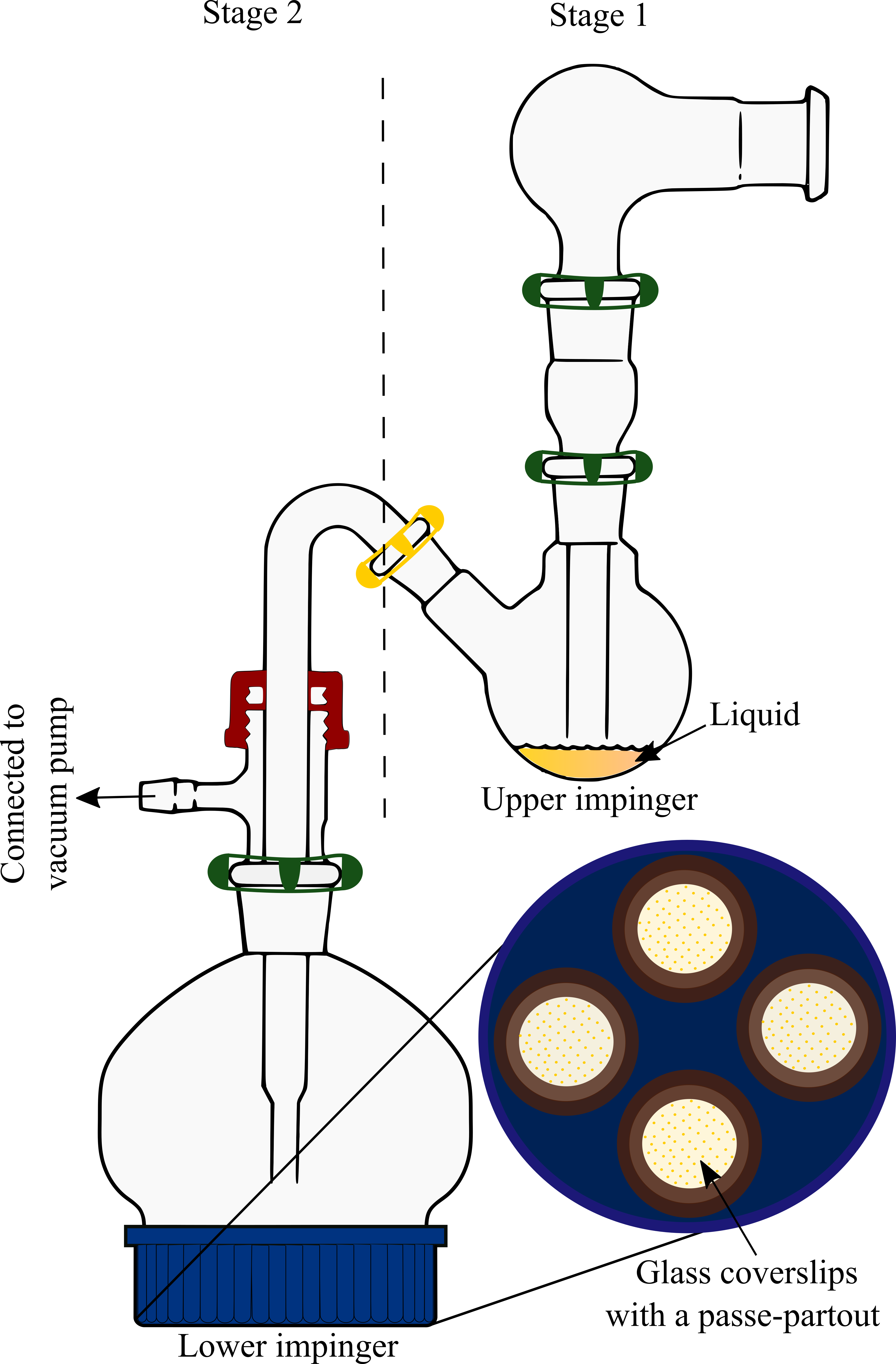
**

**Supplementary Figure S1.** Modified Twin Stage Impinger (mTSI) (reproduced from the supplementary information of Eedara et al., 2019 with the permission from Elsevier).

(Eedara, B. B. et al. Crystalline adduct of moxifloxacin with trans-cinnamic acid to reduce the aqueous solubility and dissolution rate for improved residence time in the lungs. Eur. J. Pharm. Sci. 136, 104961 (2019).)

**S1. Stella equations - dissolution of respirable size particles in a well-stirred system**

- Drug in collection tube(t) = Drug in collection tube(t - dt) + (Collection rate) * dt

INIT Drug in collection tube = 0

INFLOWS: Collection rate = Cperfusate * Flow rate

- Drug in mucus(t) = Drug in mucus(t - dt) + (Dissolution rate – Permeation rate) * dt

INIT Drug in mucus = 0

INFLOWS: Dissolution rate = Ddh * SA of particles * (Cs - Cmucus)

OUTFLOWS: Permeation rate = P * SA membrane * (C mucus - C perfusate)

- Drug in receiver(t) = Drug in receiver(t - dt) + (Permeation rate – Collection rate) * dt

INIT Drug in receiver = 0

INFLOWS: Permeation rate = P * SA membrane * (Cmucus - Cperfusate)

OUTFLOWS: Collection rate = C perfusate * Flow rate

- Solid drug particles(t) = Solid drug particles(t - dt) + (- Dissolution rate) * dt

INIT Solid drug particles = Initial mass of the drug particles

OUTFLOWS: Dissolution rate = Ddh * SA of particles * (Cs - Cmucus)

- Cs = Solubility
- C mucus = Drug in mucus/V mucus
- C perfusate = Drug in receiver/V perfusate
- Ddh = IDR/Solubility
- Diameter of particles at time t = (Solid drug particles * Initial diameter of particles^3 / Initial mass of the drug particles)^(1/3)
- Flow rate: 0.4 mL min^-1^
- hexp = 62.5× 10^-4^ cm
- hm = 62.5× 10^-4^ cm
- IDR: Moxifloxacin- 0.50 × 10^-3^ g cm^-2^ min^-1^, Ethionamide- 0.06 × 10^-3^ g cm^-2^ min^-1^
- Initial diameter of particles: Moxifloxacin- 2.9 × 10^-4^ cm, Ethionamide- 3.6 × 10^-4^ cm
- Initial mass of the drug particles: 50× 10^-6^ g
- Particle density: 1.0 g cm^-3^
- Percentage drug in collection tube = Drug in collection tube/Initial mass of the drug particles*100
- Percentage undissolved = Solid drug particles/Initial mass of the drug particles*100
- Pexp: Moxifloxacin-1.8 × 10^-4^ cm min^-1^, Ethionamide-5.3 × 10^-4^ cm min^-1^
- SA membrane: 4.91 cm^2^
- SA_of_particles = 6 * Initial mass of the drug particles * Diameter of particles at time t^2/Initial diameter of particles^3/Particle density
- Solubility: Moxifloxacin- 17.70 × 10^-3^ g cm^-3^, Ethionamide- 0.46 × 10^-3^ g cm^-3^
- Thickness of mucus = V mucus/SA membrane
- V mucus: 25 × 10^-3^ cm^3^
- V perfusate: 500 × 10^-3^ cm^3^

**S2. Stella equations - dissolution of respirable size particles in unstirred system**

- Drug in collection tube(t) = Drug in collection tube(t - dt) + (Collection rate) * dt

INIT Drug in collection tube = 0

INFLOWS: Collection rate = C perfusate*Flow rate

- Drug in mucus(t) = Drug in mucus(t - dt) + (Dissolution rate – Permeation rate) * dt

INIT Drug in mucus = 0

INFLOWS: Dissolution rate = Ddh*SA of particles*(Cs-C mucus)

OUTFLOWS: Permeation rate = P*SA membrane*(C mucus-C perfusate)

- Drug in receiver(t) = Drug in receiver(t - dt) + (Permeation rate – Collection rate) * dt

INIT Drug in receiver = 0

INFLOWS: Permeation rate = P*SA membrane*(C mucus-C perfusate)

OUTFLOWS: Collection rate = C perfusate*Flow rate

- Solid drug particles(t) = Solid drug particles(t - dt) + (- Dissolution rate) * dt

INIT Solid drug particles = Initial mass of the drug particles

OUTFLOWS: Dissolution rate = Ddh*SA of particles*(Cs-C mucus)

- Cs = Solubility
- C mucus = Drug in mucus/V mucus
- C perfusate = Drug in receiver/V perfusate
- Ddh = IDR/Solubility
- Diameter of particles at time t = (Solid drug particles*Initial diameter of particles^3 / Initial mass of the drug particles)^(1/3)
- Flow rate = 0.4 mL min^-1^
- Fraction perfused = Mass perfused/Initial mass of the drug particles
- hexp = 62.5× 10^-4^ cm
- hm = 62.5× 10^-4^ cm
- IDR: Moxifloxacin- 0.50 × 10^-3^ g cm^-2^ min^-1^, Ethionamide- 0.06 × 10^-3^ g cm^-2^ min^-1^
- Initial diameter of particles: Moxifloxacin- 2.9 × 10^-4^ cm, Ethionamide- 3.6 × 10^-4^ cm
- Initial mass of the drug particles: 50× 10^-6^ g
- Mass perfused = Drug in receiver+Drug in collection tube
- P = IF (Fraction perfused <= 0.5) THEN (Pexp*hexp/(hm+Fraction perfused*Thickness of mucus)) ELSE(Pexp*hexp/(hm+0.5*Thickness of mucus))
- Particle density: 1.0 g cm^-3^
- Percentage drug in collection tube = Drug in collection tube/Initial mass of the drug particles*100
- Percentage undissolved = Solid drug particles/Initial mass of the drug particles*100
- Pexp = Moxifloxacin-1.8 × 10^-4^ cm min^-1^, Ethionamide-5.3 × 10^-4^ cm min^-1^
- SA membrane = 4.91 cm^2^
- SA of particles = 6*Initial mass of the drug particles*Diameter of particles at time t^2/Initial diameter of particles^3/Particle density
- Solubility: Moxifloxacin-17.70 × 10^-3^ g cm^-3^, Ethionamide- 0.46 × 10^-3^ g cm^-3^
- Thickness of mucus = V mucus/SA membrane
- V mucus: 25 × 10^-3^ cm^3^
- V perfusate: 500 × 10^-3^ cm^3^

**S3. A STELLA simulation model for permeation of drug from a solution through the membrane under well-stirred conditions**

**Supplementary Figure S2.** A STELLA model for permeation of drug from a solution through the membrane under well-stirred conditions. (P_exp_, experimental permeation rate, SA, surface area, C, concentration, V, volume).

Following are the steps involved in a STELLA model for the permeation of drug through the membrane under well-stirred conditions.

- Drug in collection tube(t) = Drug in collection tube(t - dt) + (Collection rate) * dt

INIT Drug in collection tube = 0

INFLOWS: Collection rate = C perfusate*Flow rate

- Drug in mucus(t) = Drug in mucus(t - dt) + (- Permeation rate) * dt

INIT Drug in mucus = Initial mass of the drug in mucus= 50× 10^-6^ g

OUTFLOWS: Permeation rate = P*SA membrane*(C mucus-C perfusate)

- Drug in receiver(t) = Drug in receiver(t - dt) + (Permeation rate – Collection rate) * dt

INIT Drug in receiver = 0

INFLOWS: Permeation rate = P*SA membrane*(C mucus-C perfusate)

OUTFLOWS: Collection rate = C perfusate*Flow rate

- C mucus = Drug in mucus/V mucus
- C perfusate = Drug in receiver/V perfusate
- Flow rate = 0.4 mL min^-1^
- hexp = 62.5× 10^-4^ cm
- hm = 62.5× 10^-4^ cm
- Initial mass of the drug in mucus: 50× 10^-6^ g
- P = (Pexp*hexp)/hm
- Percentage drug in collection tube = Drug in collection tube/Initial mass of the drug in mucus*100
- Pexp = Moxifloxacin-1.8 × 10^-4^ cm min^-1^, Ethionamide-5.3 × 10^-4^ cm min^-1^
- SA membrane = 4.91 cm^2^
- Thickness of mucus = V mucus/SA membrane
- V mucus: 25 × 10^-3^ cm^3^
- V perfusate: 500 × 10^-3^ cm^3^

**S4. A STELLA simulation model for permeation of drug from a solution through the membrane under unstirred conditions**

**Supplementary Figure S3.** A STELLA model for permeation of drug from a solution through the membrane under unstirred conditions. (P_exp_, experimental permeation rate, P, permeation rate at time t, hm, thickness of the membrane, hexp, thickness of the membrane used in the experimental study, SA, surface area, C, concentration, V, volume).

Following are the steps involved in a STELLA model for the permeation of drug through the membrane under unstirred conditions.

- Drug in collection tube(t) = Drug in collection tube(t - dt) + (Collection rate) * dt

INIT Drugin collection tube = 0

INFLOWS: Collection rate = C perfusate*Flow rate

- Drug in mucus(t) = Drug in mucus(t - dt) + (- Permeation rate) * dt

INIT Drug in mucus = Initial mass of the drug in mucus

OUTFLOWS: Permeation rate = P*SA membrane*(C mucus-C perfusate)

- Drug in receiver(t) = Drug in receiver(t - dt) + (Permeation rate – Collection rate) * dt

INIT Drug in receiver = 0

INFLOWS: Permeation rate = P*SA membrane*(C mucus-C perfusate)

OUTFLOWS: Collection rate = C perfusate*Flow rate

- C mucus = Drug in mucus/V mucus
- C perfusate = Drug in receiver/V perfusate
- Flow rate = 0.4
- Fraction perfused = Mass perfused/Initial mass of the drug in mucus
- hexp = 62.5× 10^-4^ cm
- hm = 62.5× 10^-4^ cm
- Initial mass of the drug particles: 50× 10^-6^ g
- Mass perfused = Drug in receiver+Drug in collection tube
- P = IF (Fraction perfused <= 0.5) THEN (Pexp*hexp/(hm+Fraction perfused*Thickness of mucus)) ELSE(Pexp*hexp/(hm+0.5*Thickness of mucus))
- Percentage drug in collection tube = Drug in collection tube/Initial mass of the drug in mucus*100
- Pexp = Moxifloxacin-1.8 × 10^-4^ cm min^-1^, Ethionamide-5.3 × 10^-4^ cm min^-1^
- SA membrane = 4.91 cm^2^
- Thickness of mucus = V mucus/SA membrane
- V mucus: 25 × 10^-3^ cm^3^
- V perfusate: 500 × 10^-3^ cm^3^

**Supplementary Table S1.** Initial parameter values used for simulation.

| Parameters | Moxifloxacin | Ethionamide |
| --- | --- | --- |
| Initial mass of the drug in mucus | 50 × 10^-6^ g | 50 × 10^-6^ g |
| Experimental permeability coefficient (P_exp_) | 1.8 × 10^-4^ cm min^-1^ | 5.3 × 10^-4^ cm min^-1^ |
| Mucus simulant | 1.5% w/v PEO in PBS, pH 7.4 | |
| Mucus simulant volume | 25 × 10^-3^ cm^3^ | |
| Perfusate, its pH and flow rate | PBS, pH 7.4, 0.4 cm^3^ min^-1^ | |
| Perfusate volume in the receptor | 500 × 10^-3^ cm^3^ | |
| Surface area of the membrane | 4.91 cm^2^ | |
| *h_exp_*^*^ | 62.5 × 10^-4^ cm (hydrated membrane) | |
| *h_m_* (assumed) | 62.5 × 10^-4^ cm | |

^*^ Thickness of the membrane used in the experimental study

All the parameters were obtained from experimental results published by Eedara et al (2019)**.**

**Supplementary Table S2.** Concentrations of moxifloxacin in mucus and perfusate at various perfusate flow rates (0.2, 0.4, and 0.8 mL min^-1^) during in vitro dissolution testing of moxifloxacin respirable size particles using STELLA simulation model under well-stirred condition.

| Time (min) | C_mucus_ (g mL^-1^) | | | C_perfusate_ (g mL^-1^) | | |
| --- | --- | --- | --- | --- | --- | --- |
|  | 0.2  **(**mL min^-1^**)** | 0.4  **(**mL min^-1^**)** | 0.8  **(**mL min^-1^**)** | 0.2  **(**mL min^-1^**)** | 0.4  **(**mL min^-1^**)** | 0.8  **(**mL min^-1^**)** |
| 2 | 1.9 × 10^-6^ | 1.9 × 10^-6^ | 1.9 × 10^-6^ | 4.6 × 10^-9^ | 3.3 ×10^-9^ | 2.0 ×10^-9^ |
| 4 | 1.7 × 10^-6^ | 1.7 × 10^-6^ | 1.7 × 10^-6^ | 6.4 × 10^-9^ | 3.8 ×10^-9^ | 2.0 ×10^-9^ |
| 6 | 1.6 × 10^-6^ | 1.6 × 10^-6^ | 1.6 × 10^-6^ | 6.9 × 10^-9^ | 3.7 ×10^-9^ | 1.8 ×10^-9^ |
| 8 | 1.5 × 10^-6^ | 1.5 × 10^-6^ | 1.5 × 10^-6^ | 6.9 × 10^-9^ | 3.5 ×10^-9^ | 1.7 ×10^-9^ |
| 10 | 1.4 × 10^-6^ | 1.4 × 10^-6^ | 1.4 × 10^-6^ | 6.6 × 10^-9^ | 3.2 ×10^-9^ | 1.6 ×10^-9^ |
| 15 | 1.2 × 10^-6^ | 1.2 × 10^-6^ | 1.2 × 10^-6^ | 5.7 × 10^-9^ | 2.7 ×10^-9^ | 1.3 ×10^-9^ |
| 20 | 9.9 × 10^-7^ | 9.9 × 10^-7^ | 9.9 × 10^-7^ | 4.8 × 10^-9^ | 2.3 ×10^-9^ | 1.1 ×10^-9^ |
| 25 | 8.3 × 10^-7^ | 8.3 × 10^-7^ | 8.3 × 10^-7^ | 4.0 × 10^-9^ | 1.9 ×10^-9^ | 9.4 × 10^-10^ |
| 30 | 7.0 × 10^-7^ | 7.0 × 10^-7^ | 6.9 × 10^-7^ | 3.4 × 10^-9^ | 1.6 ×10^-9^ | 7.8 × 10^-10^ |
| 45 | 4.1 × 10^-7^ | 4.1 × 10^-7^ | 4.1 × 10^-7^ | 2.0 × 10^-9^ | 9.5 × 10^-10^ | 4.6 × 10^-10^ |
| 60 | 2.4 × 10^-7^ | 2.4 × 10^-7^ | 2.4 × 10^-7^ | 1.2 × 10^-9^ | 5.6 × 10^-10^ | 2.7 × 10^-10^ |
| 90 | 8.4 × 10^-8^ | 8.4 × 10^-8^ | 8.3 × 10^-8^ | 4.1 × 10^-10^ | 1.9 × 10^-10^ | 9.4 × 10^-11^ |
| 120 | 2.9 × 10^-8^ | 2.9 × 10^-8^ | 2.9 × 10^-8^ | 1.4 × 10^-10^ | 6.7 × 10^-11^ | 3.3 × 10^-11^ |

**Supplementary Table S3.** Concentrations of moxifloxacin in mucus and perfusate at various perfusate flow rates (0.2, 0.4, and 0.8 mL min^-1^) during in vitro dissolution testing of moxifloxacin respirable size particles using STELLA simulation model under unstirred condition.

| Time (min) | C_mucus_ (g mL^-1^) | | | C_perfusate_ (g mL^-1^) | | |
| --- | --- | --- | --- | --- | --- | --- |
|  | 0.2  **(**mL min^-1^**)** | 0.4  **(**mL min^-1^**)** | 0.8  **(**mL min^-1^**)** | 0.2  **(**mL min^-1^**)** | 0.4  **(**mL min^-1^**)** | 0.8  **(**mL min^-1^**)** |
| 2 | 1.9 × 10^-6^ | 1.9 × 10^-6^ | 1.9 × 10^-6^ | 4.4 × 10^-9^ | 3.2 × 10^-9^ | 1.9 ×10^-9^ |
| 4 | 1.8 × 10^-6^ | 1.8 × 10^-6^ | 1.8 × 10^-6^ | 6.1 × 10^-9^ | 3.6 × 10^-9^ | 1.8 ×10^-9^ |
| 6 | 1.7 × 10^-6^ | 1.7 × 10^-6^ | 1.7 × 10^-6^ | 6.4 × 10^-9^ | 3.4 × 10^-9^ | 1.6 ×10^-9^ |
| 8 | 1.6 × 10^-6^ | 1.6 × 10^-6^ | 1.6 × 10^-6^ | 6.2 × 10^-9^ | 3.1 × 10^-9^ | 1.5 ×10^-9^ |
| 10 | 1.5 × 10^-6^ | 1.5 × 10^-6^ | 1.5 × 10^-6^ | 5.8 × 10^-9^ | 2.8 × 10^-9^ | 1.4 ×10^-9^ |
| 15 | 1.3 × 10^-6^ | 1.3 × 10^-6^ | 1.3 × 10^-6^ | 4.8 × 10^-9^ | 2.3 × 10^-9^ | 1.1 ×10^-9^ |
| 20 | 1.1 × 10^-6^ | 1.1 × 10^-6^ | 1.1 × 10^-6^ | 4.0 × 10^-9^ | 1.9 × 10^-9^ | 9.2 × 10^-10^ |
| 25 | 9.8 × 10^-7^ | 9.8 × 10^-7^ | 9.8 × 10^-7^ | 3.3 × 10^-9^ | 1.6 × 10^-9^ | 7.8 × 10^-10^ |
| 30 | 8.7 × 10^-7^ | 8.6 × 10^-7^ | 8.6 × 10^-7^ | 2.9 × 10^-9^ | 1.4 × 10^-9^ | 6.9 × 10^-10^ |
| 45 | 5.9 × 10^-7^ | 5.9 × 10^-7^ | 5.9 × 10^-7^ | 2.0 × 10^-9^ | 9.6 × 10^-10^ | 4.7 × 10^-10^ |
| 60 | 4.1 × 10^-7^ | 4.1 × 10^-7^ | 4.1 × 10^-7^ | 1.4 × 10^-9^ | 6.6 × 10^-10^ | 3.2 × 10^-10^ |
| 90 | 1.9 × 10^-7^ | 1.9 × 10^-7^ | 1.9 × 10^-7^ | 6.4 × 10^-10^ | 3.1 × 10^-10^ | 1.5 × 10^-10^ |
| 120 | 9.1 × 10^-8^ | 9.0 × 10^-8^ | 9.0 × 10^-8^ | 3.0 × 10^-10^ | 1.5 × 10^-10^ | 7.2 × 10^-11^ |

**Supplementary Table S4.** Concentrations of ethionamide in mucus and perfusate at various perfusate flow rates (0.2, 0.4, and 0.8 mL min^-1^) during in vitro dissolution testing of ethionamide respirable size particles using STELLA simulation model under well-stirred condition.

| Time (min) | C_mucus_ (g mL^-1^) | | | C_perfusate_ (g mL^-1^) | | |
| --- | --- | --- | --- | --- | --- | --- |
|  | 0.2  **(**mL min^-1^**)** | 0.4  **(**mL min^-1^**)** | 0.8  **(**mL min^-1^**)** | 0.2  **(**mL min^-1^**)** | 0.4  **(**mL min^-1^**)** | 0.8  **(**mL min^-1^**)** |
| 2 | 4.5 × 10^-7^ | 4.5 × 10^-7^ | 4.5 × 10^-7^ | 2.9 × 10^-9^ | 2.2 × 10^-9^ | 1.7 × 10^-9^ |
| 4 | 4.5 × 10^-7^ | 4.5 × 10^-7^ | 4.5 × 10^-7^ | 4.5 × 10^-9^ | 2.7 × 10^-9^ | 1.9 × 10^-9^ |
| 6 | 4.5 × 10^-7^ | 4.5 × 10^-7^ | 4.5 × 10^-7^ | 5.2 × 10^-9^ | 2.9 × 10^-9^ | 1.9 × 10^-9^ |
| 8 | 4.4 × 10^-7^ | 4.4 × 10^-7^ | 4.4 × 10^-7^ | 5.5 × 10^-9^ | 2.9 × 10^-9^ | 1.9 × 10^-9^ |
| 10 | 4.4 × 10^-7^ | 4.4 × 10^-7^ | 4.4 × 10^-7^ | 5.6 × 10^-9^ | 2.9 × 10^-9^ | 1.9 × 10^-9^ |
| 15 | 4.4 × 10^-7^ | 4.4 × 10^-7^ | 4.4 × 10^-7^ | 5.7 × 10^-9^ | 2.9 × 10^-9^ | 1.9 × 10^-9^ |
| 20 | 4.4 × 10^-7^ | 4.4 × 10^-7^ | 4.4 × 10^-7^ | 5.6 × 10^-9^ | 2.8 × 10^-9^ | 1.9 × 10^-9^ |
| 25 | 4.3 × 10^-7^ | 4.3 × 10^-7^ | 4.3 × 10^-7^ | 5.6 × 10^-9^ | 2.8 × 10^-9^ | 1.9 × 10^-9^ |
| 30 | 4.2 × 10^-7^ | 4.2 × 10^-7^ | 4.2 × 10^-7^ | 5.5 × 10^-9^ | 2.7 × 10^-9^ | 1.8 × 10^-9^ |
| 45 | 1.6 × 10^-7^ | 1.6 × 10^-7^ | 1.6 × 10^-7^ | 2.7 × 10^-9^ | 1.2 × 10^-9^ | 7.4 × 10^-10^ |
| 60 | 3.5 × 10^-8^ | 3.4 × 10^-8^ | 3.3 × 10^-8^ | 6.0 × 10^-10^ | 2.5 × 10^-10^ | 1.6 × 10^-10^ |
| 90 | 1.6 × 10^-9^ | 1.5 × 10^-9^ | 1.5 × 10^-9^ | 2.8 × 10^-11^ | 1.1 × 10^-11^ | 7.0 × 10^-12^ |
| 120 | 7.5 × 10^-11^ | 6.8 × 10^-11^ | 6.6 × 10^-11^ | 1.3 × 10^-12^ | 5.0 × 10^-13^ | 3.1 × 10^-13^ |

**Supplementary Table S5.** Concentrations of ethionamide in mucus and perfusate at various perfusate flow rates (0.2, 0.4, and 0.8 mL min^-1^) during in vitro dissolution testing of ethionamide respirable size particles using STELLA simulation model under unstirred condition.

| Time (min) | C_mucus_ (g mL^-1^) | | | C_perfusate_ (g mL^-1^) | | |
| --- | --- | --- | --- | --- | --- | --- |
|  | 0.2  (mL min^-1^) | 0.4  (mL min^-1^) | 0.8  (mL min^-1^) | 0.2  (mL min^-1^) | 0.4  (mL min^-1^) | 0.8  (mL min^-1^) |
| 2 | 4.5 × 10^-7^ | 4.5 × 10^-7^ | 4.5 × 10^-7^ | 2.9 × 10^-9^ | 2.1 × 10^-9^ | 1.3 ×10^-9^ |
| 4 | 4.5 × 10^-7^ | 4.5 × 10^-7^ | 4.5 × 10^-7^ | 4.3 × 10^-9^ | 2.6 × 10^-9^ | 1.4 ×10^-9^ |
| 6 | 4.5 × 10^-7^ | 4.5 × 10^-7^ | 4.5 × 10^-7^ | 4.8 × 10^-9^ | 2.6 × 10^-9^ | 1.3 ×10^-9^ |
| 8 | 4.5 × 10^-7^ | 4.5 × 10^-7^ | 4.5 × 10^-7^ | 5.0 × 10^-9^ | 2.6 × 10^-9^ | 1.3 ×10^-9^ |
| 10 | 4.5 × 10^-7^ | 4.5 × 10^-7^ | 4.5 × 10^-7^ | 5.0 × 10^-9^ | 2.5 × 10^-9^ | 1.2 ×10^-9^ |
| 15 | 4.5 × 10^-7^ | 4.5 × 10^-7^ | 4.5 × 10^-7^ | 4.7 × 10^-9^ | 2.3 × 10^-9^ | 1.2 ×10^-9^ |
| 20 | 4.4 × 10^-7^ | 4.4 × 10^-7^ | 4.4 × 10^-7^ | 4.5 × 10^-9^ | 2.2 × 10^-9^ | 1.1 ×10^-9^ |
| 25 | 4.4 × 10^-7^ | 4.4 × 10^-7^ | 4.4 × 10^-7^ | 4.2 × 10^-9^ | 2.1 × 10^-9^ | 1.0 ×10^-9^ |
| 30 | 4.4 × 10^-7^ | 4.4 × 10^-7^ | 4.4 × 10^-7^ | 4.1 × 10^-9^ | 2.0 × 10^-9^ | 1.0 ×10^-9^ |
| 45 | 3.9 × 10^-7^ | 3.9 × 10^-7^ | 3.9 × 10^-7^ | 3.7 × 10^-9^ | 1.8 × 10^-9^ | 9.1 × 10^-10^ |
| 60 | 1.4 × 10^-7^ | 1.4 × 10^-7^ | 1.4 × 10^-7^ | 1.6 × 10^-9^ | 7.0 × 10^-10^ | 3.3 × 10^-10^ |
| 90 | 1.6 × 10^-8^ | 1.5 × 10^-8^ | 1.5 × 10^-8^ | 1.7× 10^-10^ | 7.6 × 10^-11^ | 3.6 × 10^-11^ |
| 120 | 1.7 × 10^-9^ | 1.7 × 10^-9^ | 1.6 × 10^-9^ | 1.9 × 10^-11^ | 8.4 × 10^-12^ | 3.9 × 10^-12^ |
